# Seed Biology of *Lepidium apetalum* (Brassicaceae), with Particular Reference to Dormancy and Mucilage Development

**DOI:** 10.3390/plants9030333

**Published:** 2020-03-05

**Authors:** Keliang Zhang, Yin Zhang, Yusong Ji, Jeffrey L. Walck, Jun Tao

**Affiliations:** 1 Jiangsu Key Laboratory of Crop Genetics and Physiology, College of Horticulture and Plant Protection, Yangzhou University, Yangzhou 225009, China; zhangkeliang@yzu.edu.cn (K.Z.);; 2 Department of Biology, Middle Tennessee State University, Murfreesboro, TN 37132, USA

**Keywords:** germination, *Lepidium apetalum*, mucilage, osmotic potential, seed coat, seedling growth

## Abstract

*Lepidium apetalum* (Brassicaceae) is an annual or biennial weed widely distributed in Asia and Europe. The outer surface of *L. apetalum* seeds produces a large amount of mucilage. The primary aim of this study was to explore the dormancy characteristics and to determine how mucilage develops. The role of mucilage in water absorption/dehydration, the effects of after-ripening, gibberellin acid (GA_3_), cold stratification and seed coat scarification on germination, the role of mucilage in germination and seedling growth during drought, and the progress of mucilage production during seed development were investigated. The results indicate that the best temperature regime for germination was 10/20 °C. After-ripening, GA_3_ and seed coat scarification helped to break dormancy. Light promoted germination. Seedling growth of mucilaged seeds were significantly higher than those of demucilaged seeds at −0.606 and −1.027 MPa. Anatomical changes during seed development showed that mucilage was derived from the outer layer of the outer integument cells. Our findings suggest that seeds of *L. apetalum* exhibited non-deep physiological dormancy. The dormancy characteristics along with mucilage production give seeds of *L. apetalum* a competitive advantage over other species, and thus contribute to its potential as a weed. Effective control of this weed can be achieved by deep tillage.

## 1. Introduction

Heterogeneous environments create natural selection pressure that results in variations in plant growth and reproduction [1]. Each species of plant responds uniquely to environmental conditions during growth and over time the species evolves; many strategies enhance a species’ ability to adapt rapidly in response to changes in the environment [2,3]. Seed dormancy is an important part of a plant’s life history and strategy and can prevent seeds from germinating when the environment is unsuitable for seedling survival [2,4]. The timing of seed germination determines the survival, growth and reproduction of plants, affecting species persistence and distribution, which is directly related to each species’ population and to the composition, structure and dynamics of communities [5,6].

Diverse mechanisms and processes are involved in seed dormancy which allows plants to adapt to a diversity of climates and habitats in which they grow [5]. The key to understanding the germination of seeds is to identify what kind of environmental signals can overcome dormancy. In addition to the basic requirement for temperature, water and oxygen, a dormant seed may also be sensitive to other factors, such as hormones, light and pH [2,5,6]. Moreover, environmental conditions can also interact with biological aspects of seeds, such as mucilage production, to control germination. Mucilage is usually a composite of cellulosic, non-cellulosic, and pectic polysaccharides [7,8,9]. As mucilage absorbs water, it swells and covers the seed with jelly-like layers [9,10]. It has been reported to be produced by seeds or fruits of species in 37 orders, 110 families and at least 230 genera of angiosperms, especially in Cruciferae, Asteraceae, Boraginaceae, Lamiaceae, Acanthaceae, and Scrophulariaceae [2,10].

The presence of mucilage on the seed coat is believed to provide a favorable environment at different stages of seed development and to promote the maturation of seeds before the rainy season so that the seeds can germinate when rainfall arrives [11,12,13]. In addition, mucilage also prevents seeds from completely drying out by retaining moisture [14,15] and increases the contact area of seeds and soil, which promotes the growth of the seedlings [16,17]. Seed mucilage enhances water uptake during germination due to its hygroscopic properties [10]. Mucilage also aids in fruit and seed dispersal and defend against pathogens; however, it can delay germination by impeding diffusion of oxygen [10,18].

*Lepidium apetalum* Wild, a member of Cruciferae, is an annual or biennial weed with the height of 10–25 cm, flowering and fruiting from April to June [19]. This species is distributed throughout Asia and Europe [19,20,21]. Its natural habitats include roadsides, slopes, ravines, cultivated fields, and other human disturbed habitats [19]. Modern pharmacological studies have shown that seeds of *L. apetalum* have anti-oxidant, antibacterial, and cardiac activities [21,22]. Moreover, the seeds contained various secondary metabolites, such as oil, flavonoids, sterols and cardiac glycosides making it an important resource to develop and take advantage of [21,22].

Seed propagation is the only mechanism by which *Lepidium* species regenerate [23,24,25]. A previous study showed that fresh mature seeds of *L. perfoliatum* and *L. virginicum* were dormant and dry storage at 30 °C for four months [23] and −18 °C [25] broke dormancy, respectively. After dormancy was broken, seeds of *L. perfoliatum* germinated at 0 °C while those of *L. apetalum* could not [24]. However, the dormancy characteristics of *L. apetalum* seeds and the regeneration strategies of this species heretofore have not been investigated; understanding its germination ecology may be useful for weed control. Moreover, seeds of *L. apetalum* contain a high proportion of mucilage. It remains unknown how mucilage of *L. apetalum* affects seed germination and seedling establishment.

What makes *L. apetalum* a weed species? The following questions are raised: (1) are *L. apetalum* seeds dormant and how to break? (2) can seed mucilage promote germination of *L. apetalum* seeds? (3) can mucilage promote early seedling growth under drought conditions? and (4) how does mucilage develop? To solve those questions, we determined the (1) change in seed characteristics with mucilage production, (2) water absorption and dehydration characteristics, (3) effects of temperature, light, and/or gibberellic acid (GA_3_) on germination of fresh and dry-stored seeds, (4) effects of cold stratification on germination, (5) effects of seed coat scarification on germination, (6) role of mucilage in germination and seedling growth during droughts, and (7) developmental progress of mucilage production during seed development. The results from these experiments will improve our understanding of the ecological roles of seed mucilage in life history strategies and will help to develop effective measures for controlling *L. apetalum*.

## 2. Results

### 2.1. Seed Characteristics

Freshly matured seeds of *L. apetalum* were reddish brown, long-oval in shape. During imbibition, the production of mucilage in the seed coat absorbed a large amount of water and length (*F* = 227.656, *P* < 0.001), width (*F* = 14.405, *P* = 0.005), and 1000-seed weight (*F* = 8638.087, *P* < 0.001) of imbibed seeds were significantly greater than they were for fresh seeds (Table 1).

### 2.2. Water Absorption and Dehydration Characteristics

**Figure 1:**
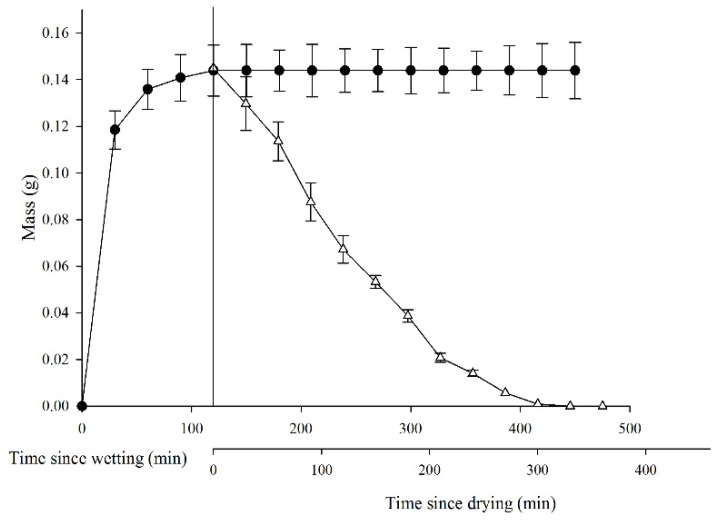
Dynamics of water absorption and dehydration in *Lepidium apetalum* seeds. ● represents water absorption of mucilaginous seeds; Δ represents dehydration of mucilaginous seeds. Values represent mean ± SE.

Seeds of *L. apetalum* imbibed water quickly, and seed mass increased by 0.118 ± 0.008 in 30 min and by 0.143 ± 0.09 in 120 min, at which time water imbibition had reached its maximum (Figure 1). By contrast, the dehydration process was relatively slow, seeds retained 0.071 ± 0.06 g water for 120 min and did not return to their original masses until 360 min.

### 2.3. Effects of Temperature, Light, and/or GA_3_ on Germination of Fresh and Dry-Stored Seeds

**Figure 2:**
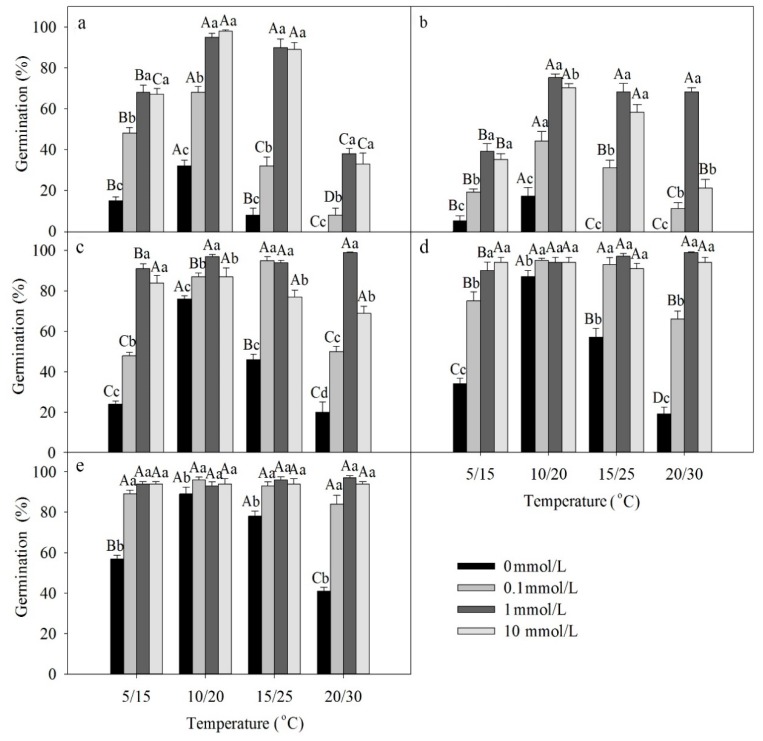
Effects of GA_3_ and temperature on germination percentage of seeds of *Lepidium apetalum* (mean ± SE) on germination of (**a**) freshly matured seeds in light, (**b**) freshly matured seeds in darkness, (**c**) 1-month dry stored seeds in light, (**d**) 2-month dry stored seeds in light, and (**e**) 3-month dry stored seeds in light. Different uppercase letters indicate significant differences among temperatures (within a GA_3_ concentration) and lowercase letters indicate differences among GA_3_ concentration (within a temperature) (5% level).

A three-way ANOVA showed that temperature, light, GA_3_ and the interactions among these factors had significant effects on germination of freshly matured seeds (Table 2). Germination was higher in light than darkness. The best temperature regime for germination of freshly matured seeds was 10/20 °C (Figure 2a,b). GA_3_ significantly increased germination of fresh seeds at all four temperature regimes in both light and darkness.

Dormant seeds gradually after-ripened during storage. Three-way ANOVA results showed that temperature, GA_3_ and dry storage time, as well as all 2- and 3-way interactions among these factors had significant effects on germination of dry-stored seeds (Table 3). As the time in dry storage increased, germination in light and over the GA_3_ gradient increased to moderate to high percentages (compare Figure 2a to Figure 2c–e).

### 2.4. Effects of Cold Stratification on Germination

Despite the length of cold stratification, the seeds germinated at 10/20 °C had the highest germination percentages, but only <40% of seeds germinated. A two-way ANOVA indicated that the germination percentage was significantly affected by temperature (*F* = 99.997, *P* < 0.001), but not by cold stratification duration (*F* = 2.275, *P* = 0.072) and the interaction of them (*F* = 0.997, *P* = 0.463) (figure not shown).

### 2.5. Scarification of the Seed Coat

**Figure 3:**
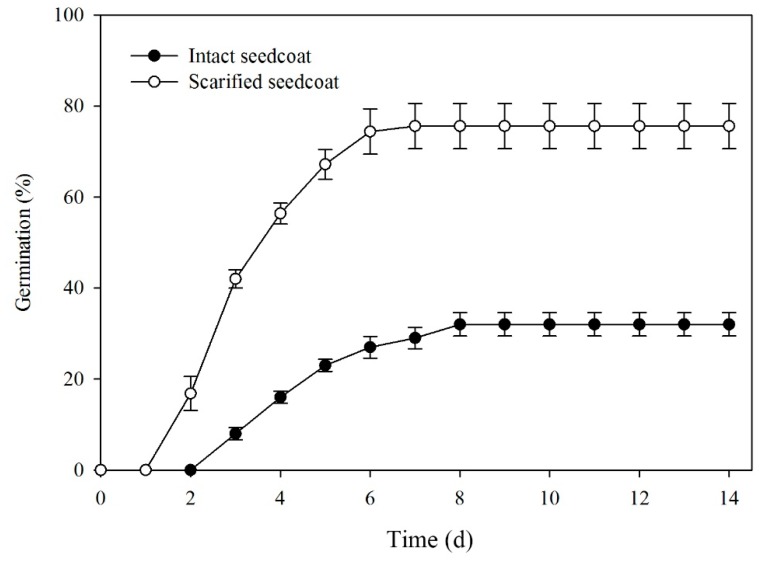
Mean (±SE) germination percentages of manually scarified and intact seeds of *Lepidium apetalum* incubated at 10/20 °C.

Scarifying the seed coat significantly increased germination at 10/20 °C (*F* = 52.920, *P* < 0.001). Germination of intact seeds was 34%, while that of scarified seeds was 76% after 8 days of incubation and percentages remained unchanged up to 14 d (Figure 3).

### 2.6. Effects of PEG on Germination and Seedlings of Mucilaged and Demucilaged Seeds

**Figure 4:**
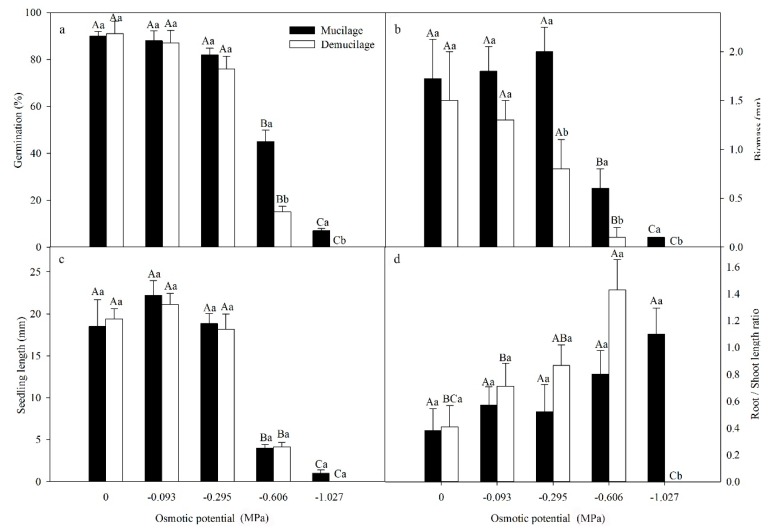
Mean (±SE) germination percentages at 30 days (**a**) and biomass (**b**), length (**c**) and root/shoot length ratio (**d**) of seedlings at 15 days of *Lepidium apetalum*. Seeds either contained mucilage or did not (demucilage) and both seed types were incubated in the different osmotic potentials in light at 20 °C. The different uppercase letters indicate significant differences among osmotic potentials and the different lowercase letters significant differences between mucilaged and demucilaged seeds within each osmotic potential (5% level).

A two-way ANOVA showed that seed germination was significantly affected by the presence or absence of mucilage (*F* = 83.922, *P* < 0.001), osmotic potential (*F* = 191.599, *P* < 0.001), and their interaction (*F* = 15.93, *P* < 0.001). With the increase of the osmotic potential of PEG, germination percentages decreased (Figure 4a). Germination of mucilaged seeds was significantly higher than that of demucilaged seeds at -0.606 and −1.027 MPa (Figure 4a). Seedling biomass and seedling length decreased with increased osmotic potentials and the mucilaged seeds had higher biomass than demucilaged seeds at −0.295, −0.606 and −1.027 MPa (Figure 4b).

A two-way ANOVA indicated that seedling biomass was significantly affected by PEG concentration (*F* = 1429.833, *P* < 0.001), mucilage presence/absence (*F* =88.048, *P* < 0.001) and their interaction (*F* = 36.738, *P* < 0.001). However, compared with biomass, seedling length of mucilaged and demucilaged seeds did not differ. A two-way ANOVA indicated that seedling length was significantly affected by PEG concentration (*F* = 82.874, *P* < 0.001), but not by mucilage presence/absence (*F* = 0.137, *P* = 0.714) and their interaction (*F* = 0.172, *P* = 0.951).

Root/shoot length ratios increased with increased osmotic potentials (Figure 4d). The root/shoot length ratio of demucilaged seeds was higher than mucilaged seeds at −0.606 MPa. A two-way ANOVA indicated that root/shoot length ratio was significantly affected by mucilage (*F* = 5.055, *P* = 0.003) and the interaction of mucilage and PEG concentration (*F* = 7.642, *P* < 0.001), but not by the presence or absence of mucilage (*F* = 0.007, *P* = 0.932).

### 2.7. Anatomical Changes of Mucilage During Seed Development

**Figure 5:**
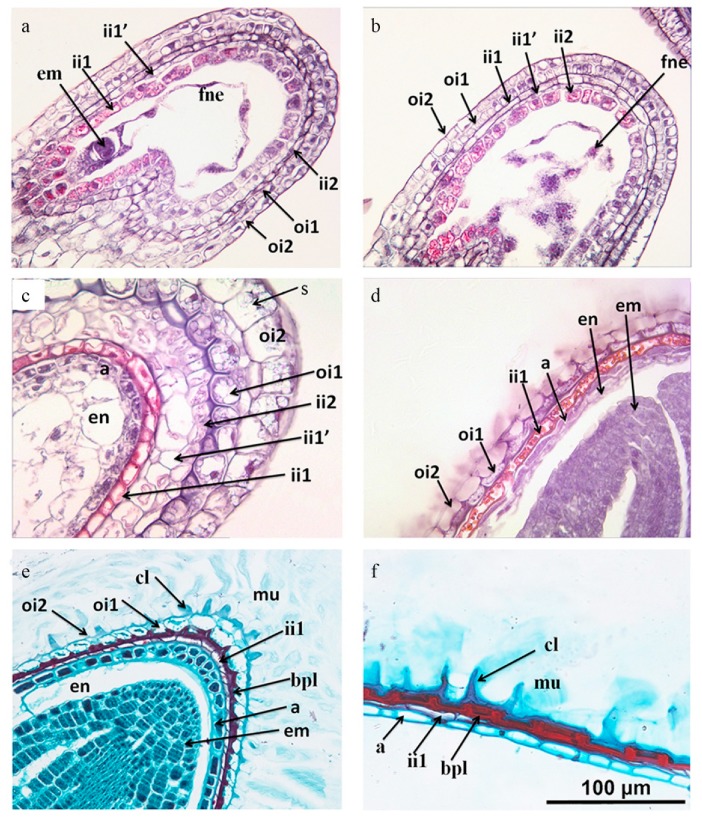
Longitudinal sections of developing seeds of *Lepidium apetalum*. (**a**) One-cell stage. (**b**) Two-cell stage. (**c**) Torpedo embryo stage, numerous starch granules appears in the layers of the outer integument, the periclinal cell wall of oil layer is thickened, aleurone layer is formed. (**d**) Bent-cotyledon stage, ii1′ and ii2 cells and cellular endosperm become crushed, aleurone layer becomes distinguishable, endosperm layer, except for the outermost layers, becomes gradually consumed by the growing embryo; (**e**) Mature embryo stage. The oi1 layer is still discernible by its thickened inner periclinal cell walls, forming a brown pigment layer. The oi2 layer is differentiated into the surface cell of the seed coat and the columella is formed. (**f**) Mature seeds with the oi1 layer largely crushed. a, aleurone; bpl, brown pigment layer; cl, columella; em, embryo; en, endosperm; fne, free nuclear endosperm; ii, inner integument; ii1, inner epidermis of inner integument; ii1′, median layer of inner integument; ii2, outer layer of inner integument; mu, mucilage; oi, outer integument; oi1: inner epidermis of outer integument; oi2, outer epidermis of outer integument; s, starch grain.

The longitudinal sections through the embryo indicated that the ovule is campylotropous. The seed coat consisted of five layers of special cell types originating from two integuments (Figure 5a). The outer integument (oi) consisted of two cell layers, an inner (oil) cell layer and an outer (oi2) layer, both produced starch grains (Figure 5b) and then degraded (Figure 5c). The oi2 layer differentiated into the surface cell of the seed coat, which is called columellae and contained mucilage. From the early stage until the mature embryo stage, the oil layer remained discernible by its thickened inner periclinal walls (Figure 5c–f). At the desiccation stage, this layer mostly collapsed but the inner periclinal cell wall contributed to the brown pigment layer (bpl).

The inner integument cell layer was composed of three layers (ii1, ii1′ and ii2), but it only had two layers at the micropyle and chalaze region (iil and ii2) (Figure 5a). The innermost cell layer (ii1) was the endothelium. From the early stage of the embryo, the cells of the iil layer showed a remarkable change in structure. At the desiccation stage, ii1 had become a layer of empty thick-walled dead cells (Figure 5c,d–f), the iil′ and ii2 layers were collapsed, forming a brown pigment layer (bpl).

At the early stage, the endosperm was characterized by free-nuclear endosperm. With the increase in the number of nuclei, the nuclei were extruded to the embryo sac, being more intensive at micropyle and chalaza than on the lateral side of the sac where it was only one layer (Figure 5b). During the last stage, much of the endosperm was crushed and absorbed by the expanding embryo, with only the aleurone layer remaining.

## 3. Discussion

Plant species have evolved a number of morphological and physiological adaptations to increase survival and fitness [1]. *L. apetalum* is a weed and medicinal species widely distributed in Asia and Europe, therefore increasing our understanding of its germination ecology may be useful for its control. Thus, to understand *L. apetalum* strategies uses to adapt to its natural habitat, we investigated the dormancy and germination characteristics and the role of mucilage during droughts on germination of this species.

After plants of *L. apetalum* complete their life cycle in May to June, the two sides of the silique split off and the seeds are dispersed. The production of mucilage may help prevent the removal of seeds by wind from the vicinity of the mother plants, depending on the timing of rainfall in relation to the timing of the opening of the siliques [26,27,28]. After imbibition, seeds of *L. apetalum* form a granule with a layer of ephemeral sticky mucilage and they retain moisture for a relatively long time as they dehydrate (Figure 1). The formation of such large granules with seeds at their centers enables them to germinate near the mother plant, and the low rate of dehydration indicates that the mucilage on *L. apetalum* seeds can serve as a water reservoir for germination which is conducive to seedling survival in some extreme environments [28,29]. Moreover, mucilage could adhere to the soil surface and thereby prevent seeds of *L. apetalum* from being further dispersed by wind from favorable microhabitats. Mucilage also allows a seed to sink below the soil’s surface to a depth with a suitable amount of light and moisture for seed germination [10]. In addition, mucilage may prevent seed collection by consumers such as ants, thereby decreasing seed loss [28,30,31].

Since germination of freshly matured seeds of *L. apetalum* incubated under four temperature regimes in light and darkness was ≤32%, we concluded that they were dormant. Moreover, seeds of Cruciferae are water permeable and have a fully developed embryo [2]. Thus, seeds of *L. apetalum* exhibited physiological dormancy (PD). Furthermore, the promotion of germination by GA_3_, after-ripening in dry storage, and seed coat scarification indicated that these seeds have non-deep PD. Altogether, these results suggest that a large percentage of embryos in freshly matured *L. apetalum* seeds have a low growth potential and cannot break the mechanical resistance of the seed coat until dormancy is broken [2,5].

The timing of dormancy break can determine the distribution and population persistence over time [2,5]. Temperature and rainfall are two of the most important environmental factors promoting dormancy break and germination. Cold stratification has been widely used to simulate the condition that seeds of temperate-zone species experience in winter, while warm stratification is used to simulate the warm and humid environment of summer [32]. Seeds of some species require warm, moist conditions for maximum dormancy break to occur, but those of many other species will come out of dormancy while they are dry. For *L. apetalum* seeds, we found that cold stratification did not break dormancy but warm environmental conditions did so. After 3 months of after-ripening, seeds of *L. apetalum* germinated to high percentages at cool but not at warm temperatures. Warm and dry storage has also been shown to be effective in breaking dormancy of *L. perfoliatum* [23]. Since freshly matured seeds of *L. apetalum* were dormant, germination will be prevented in the summer following maturation. Hence, seeds probably germinate in nature during autumn or spring.

Since freshly matured seeds *L. apetalum* germinated to 32% in 30 days at 10/20 °C in the laboratory, it seems reasonable that at least part of a seed cohort can germinate in the field during summer. However, we never observed any seedlings of *L. apetalum* in the summer, perhaps since environmental conditions are unfavorable for germination. Other weed species that germinate mainly in autumn in nature also showed highest germination percentages at the 10/20 °C temperature regime [33,34]. Seed dormancy status is associated with seasonal temperature changes [2,5,32]. Thus, we speculate that nondormant seeds of *L. apetalum* may have entered dormancy. Dormancy induction in seeds of many species stops at conditional dormancy and thus, they lose their ability to germinate at some but not all test conditions [2,5]. Furthermore, cold stratification treatments did not promote dormancy break. Thus, germination of *L. apetalum* is prevented in winter when the habitat is cool and dry. Long-term field experiments will be needed to determine whether dormancy cycling occurs.

Seeds of *L. apetalum* had higher germination in light than in darkness, suggesting that seeds on the soil surface are capable of germinating more so than those that are buried. Light acts as an indicator of soil depth for seeds, allowing seeds near the surface to have higher germination than those deeply buried [35,36,37]. The light-requiring characteristic of *L. apetalum* seeds suggest that even though dormancy is broken, seeds can form a soil seed bank, to an extent, associated with low temperature inhibition in winter, to favor persistence of its populations in adverse environments. Therefore, deep tillage could be an effective way to minimize *L. apetalum* germination.

Water uptake is required for metabolic processes required for seed germination to be activated [5]. After seed dormancy, their sensitivity to water stress ranges from high to relatively low, depending on the species [2]. Under simulated drought conditions, germination of *L. apetalum* decreased with increasing osmotic potential of PEG, and no demucilaged seeds germinated at −1.027 MPa (Figure 4), indicating that seeds cannot germinate unless the soil is moist in the habitat. At 0, −0.093, and −0.295 MPa osmotic potentials, seed germination and seedling growth of mucilaged and demucilaged seeds did not differ. However, at −0.606 and −1.027 MPa seed germination and seedling growth were reduced for demucilaged seeds compared to mucilaged seeds. Similar results were found for *Artemsia sphaerocephala* [11] and *Alyssum minus* [38]. Given that more mucilaged seeds of *L. apetalum* germinated under increased water potential suggested that the water in the mucilage was of benefit to the germinating seed. This result also indicates that the moisture in the mucilage provided the seed additional time to germinate and provided hydration for the initial stages of seedling growth [11]. Thus, the mucilage of *L. apetalum* seeds is of benefit to the seed and the developing seedling. This may give seeds of *L. apetalum* a competitive advantage over other species and thus, contribute to its potential as a weed.

Most of the histological changes observed in *L. apetalum* (Figure 5) were in agreement with the descriptions for other members of Cruciferae, like *Arabidopsis* [39] and *Capsella bursa-pastoris* [40]. During fruit or seed development, the epidermal cells of the outer ovule integument differentiated into a specialized seed coat cell type producing extracellular pectinaceous mucilage and a volcano-shaped secondary cell wall [3,8,41]. When mature seeds come into contact with water, the mucilage absorbs moisture and expansion occurs. The periclinal and radial wall of oi2 split at the junction to complete the release of mucilage, which expands to form a gel around the seed coat. Starch accumulates before the secretion of mucilage, and it is not necessary to the formation and differentiation of seed coat nor as precursors during the formation and differentiation of mucilage. Starch formation may provide carbon and energy for mucilage production [42,43]. At the end of mucilage production, a secondary wall forms around the columnar cytoplasm and starch contracts. As the cell dehydrates, mucilage is concentrated by the outer radial wall and the outline of a small column shows. The presence of a columella increases the surface/volume ratio so that a large surface is available for mucilage deposition [9].

## 4. Materials and Methods

### 4.1. Seed Collection and Site Description

During April 2013, freshly matured and fully developed *L. apetalum* seeds were collected from natural populations at the Botanical Garden, Institute of Botany, Chinese Academy of Sciences in Beijing, China (39°59′29″N, 115°12′25″E, 82 m a.s.l.). Experiments were initiated within 2 weeks after collection to prevent changes in initial seed dormancy conditions [2].

**Figure 6:**
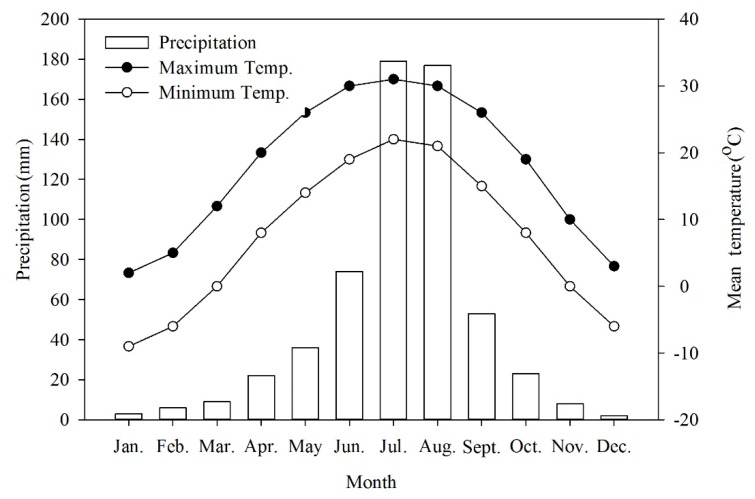
Mean precipitation and maximum and minimum temperatures in Beijing, China.

The collection site has a typical northern temperate semi-humid continental monsoon climate with hot and rainy summers, cold and dry winters, and a short spring and autumn. Nearly 30 years of climate data obtained from China Meteorological Data Sharing Service System (http://data.cma.cn/en) show that the average annual temperature was 12.3°C. Average temperatures in January and July ranged from −7 to −4 °C and from 35 to 36 °C, respectively (Figure 6). Extremes occurred from a minimum of −18.3 °C to a maximum of 41.9 °C. The annual frost-free period lasted 180 to 200 days. The average annual precipitation was 571.9 mm of which 80% fell from June to August.

### 4.2. Seed Characteristics

Eight groups each of 1000 freshly matured seeds were selected and were weighed on an analytical balance (0.0001 g) to determine seed weight (Sartorius BP 221 S, Sartorius AG, Gottingen, Germany). Seed length and width were measured on 20 seeds with a dissecting microscope (Nikon 80i, Nikon Corp., Tokyo, Japan). These same parameters were measured on the same 20 seeds after they were allowed to imbibe for 2 h.

### 4.3. Water Absorption and Dehydration Characteristics of Seeds

To test water absorption and dehydration characteristics of seeds (with mucilage left intact), 100 randomly selected seeds (25 seeds for each of four replicates) were placed in Petri dishes on a single-layer of moist filter paper, immediately weighed, and re-weighed every 30 min on the analytical balance until the seed failed to continue to absorb water. The measurements were done at room temperature (22–25 °C) and with a relative humidity of 17–30%. Then, the seeds were dried naturally under the same conditions and re-weighed once every 30 min until the initial weight of the dry seeds was re-observed. The amount of water (W) taken up for each replication of 25 seeds was calculated using Equation (1):

W = W_i_ − W_d_ (1)

where W_i_ and W_d_ are the masses of imbibed and dry seeds, respectively.

### 4.4. Effects of Temperature, Light, and/or GA_3_ on Germination of Fresh and Dry-Stored Seeds

Seeds were tested for germination when freshly matured and following dry storage at room conditions (22–25 °C, relative humidity 45–50%) for 1, 2, and 3 months. The effects of temperature, light, and/or GA_3_ were tested on fresh and dry-stored seeds. One hundred randomly selected seeds (25 seeds for each of four replicates) were used for each test. Each group of 25 seeds was placed in a 5-cm diameter Petri dish, on two layers of filter paper moistened with 2.5 mL of distilled water or GA_3_ solution. Petri dishes were sealed with parafilm.

Four temperature conditions were used for both fresh and dry-stored seeds: 5/15, 10/20, 15/25 and 20/30 °C. These temperature regimes represented the average daily minimum and maximum temperatures in Beijing, China: 5/15 °C for April and November; 10/20 °C, May; 15/25 °C, June and October; and 20/30 °C, July to September. Two light conditions were used in the experiment: 12/12 h light/dark (referred to as light) and complete darkness. Fresh seeds were tested in both light and in complete darkness, but dry-stored seeds only in light. Each dish in the complete dark treatment was placed in an opaque pocket to keep the seeds in darkness. Four concentrations of GA_3_ were tested on fresh and dry-stored seeds. The concentrations were 0 (water), 0.1, 1.0, and 10.0 mmol/L.

Seeds were considered to have germinated when the radicle protruded through the seed coat. For seeds in light, germination was scored once every 2 days, for a total of 30 days. For seeds in darkness, germination was scored only at the end of 30 days. At the end of the germination tests, non-germinating seeds were tested for viability. The seeds were incised with a scalpel and viewed under a microscope: seeds with white and hard embryos were considered viable while those with brown and soft embryos were not considered viable [26].

### 4.5. Effects of Cold Stratification on Germination

To determine the response of *L. apetalum* seeds to cold stratification, fresh seeds were placed evenly on two layers of filter paper on top of clean sand in a 20 cm diameter, 10-cm deep tin container; both the filter papers and sand were moistened with distilled water. Then, the tin was closed tightly and placed in a refrigerator at 4 °C; four sets of seeds were stratified for periods of 2, 4, 8, or 12 weeks. After each stratification period, the seeds (number and replications as above) were tested for germination at 5/15, 10/20, 15/25, and 20/30 °C in light by scoring once every 2 days for a total of 30 days.

### 4.6. Scarification of the Seed Coat

To determine the effects of the seed coat on germination, germination was tested by carefully scarifying the seed coat with a scalpel and having a control with an intact seed coat. For each condition (scarified and intact), 25 seeds were randomly selected; each test was repeated four times. Both conditions were incubated at 20/10 °C in light, because germination occurred highest at this temperature regime. The length of incubation was 14 day and seeds were scored once each day for germination. Water was added into the dishes when necessary.

### 4.7. Effects of PEG on Germination and Seedlings of Mucilaged and Demucilaged Seeds

To determine the effects of drought stress on germination of mucilaged (intact) and demucilaged seeds, polyethylene glycol 6000 (PEG; Yuanyebio Co., Shanghai, China) was used to generate five levels of osmotic stress. Four PEG solutions were formulated with distilled water: 0 (control), −0.093, −0.295, −0.606 and −1.027 MPa of PEG 6000 solution [44]. To remove the mucilage from a large number of seeds, intact seeds were submerged in water for 5 min. Then, they were removed from water and rubbed gently and rapidly on filter paper until no mucilage was released from them [11,18,45,46]. Each seed type was tested at each osmotic potential using a set of four replicates of 25 seeds each in 5 cm Petri dishes. For each dish, 2.5 mL of the corresponding solution was added and then sealed with parafilm to reduce water loss. Seeds were allowed to germinate for 15 days at 20 °C in light. At that point, the biomass, length, and root/shoot length ratio of the seedlings were measured and seeds were allowed to germinate for another 15 days, which ended the experiment.

### 4.8. Anatomical Changes of Mucilage during Seed Development

To study the development of mucilage during seed development, we examined different developmental stages from the bottom to the top of the inflorescence (racemes, flowering starts at the bottom and progresses to top). Each stage of development was immediately fixed in formaldehyde-acetic acid (FAA) fixative (a mixture of 95% ethanol, 5% acetic acid, and 5% formaldehyde). Fixative and materials ratio generally was not less than 20:1. The plant materials were placed in the fixative using a vacuum suction pump. Then, each stage was dehydrated in a series of alcohol and embedded into conventional paraffin. The plant material was sliced to 6-μm thickness using a microtome (KD 3358, Wantong Precision Instruments Corp., Zhejiang, China), stained with 1% Safranin and 2% Fast Green, and observed and photomicrographed with a light microscope (Nikon 80i, Nikon Corp., Tokyo, Japan) and digital camera (NIS-Elements D 3.0, Nikon Corp., Tokyo, Japan).

### 4.9. Data Analyses

All data are expressed as mean ± standard error and the data were analyzed for normal distribution and homogeneity of variance. If data were not normally distributed and the variances were heteroscedastic, the Kruskal–Wallis non-parametric test was used [26]. Analyses of variances (ANOVA) was used to compare seed length, width and thousand seed weight of seeds before and after seeds imbibed water, to test the effects of temperature, light regime, and GA_3_ concentration on germination of fresh seeds, to test the effects of storage time, temperature, and GA_3_ concentration on germination of dry-storaged seeds, and to test the effects of osmotic potential on germination and seedling growth between mucilaged and demucilaged seeds. Tukey’s HSD test was used for multiple comparisons among treatments (*P* < 0.01). SPSS19.0 statistical analysis software was used during data processing and analyses (SPSS Inc., Chicago, IL, USA).

## 5. Conclusions

Freshly matured seeds of *L. apetalum* exhibited non-deep physiological dormancy when dispersed in late spring and with after-ripening during warm, dry conditions, they gained the ability to germinate by late summer or early autumn. Mucilage not only increased the amount of water absorbed by seeds and decreased the rate of dehydration, but it also buffered osmotic stress, thereby promoting germination of non-dormant seeds as water stress increased. Anatomical changes during seed development showed that mucilage of *L. apetalum* was derived from the outer layer of the outer integument cells. The dormancy and germination characteristics along with mucilage production is of benefit to the seed and the developing seedling. This may give seeds of *L. apetalum* a competitive advantage over other species and thus contribute to its potential as a weed.

## Figures and Tables

**Table 1:** Comparison of the length, width, and 1000-seed mass of freshly matured and imbibed *Lepidium apetalum* seeds (mean ± se).

	Length (mm)	Width (mm)	Mass of 1000 Seeds (g)
Freshly matured seeds	1.24 ± 0.03	0.75 ± 0.33	0.21 ± 0.01
Imbibed seeds	1.82 ± 0.25	1.52 ± 0.55	1.52 ± 0.01

**Table 2:** Three-way ANOVA results testing the effects of temperature, light condition, GA_3_ and their interactions on germination of freshly matured *Lepidium apetalum* seeds. df, degree of freedom; MS, mean square; SS, sum of squares.

Source	df	SS	MS	*F*-Value	*P*-Value
Temperature	3	27325.0	9108.333	236.069	<0.001
Light	1	5724.5	5724.500	148.367	<0.001
GA_3_	3	66996.0	22332.000	578.799	<0.001
Temperature × Light	3	4356.5	1452.167	37.637	<0.001
Temperature × GA_3_	9	6373.0	708.111	18.353	<0.001
Light × GA_3_	3	1445.5	481.833	12.488	<0.001
Temperature × Light × GA_3_	9	2329.5	258.833	6.708	<0.001

**Table 3:** Three-way ANOVA results testing the effects of storage time, GA_3_, temperature and their interactions on germination of *Lepidium apetalum* seeds stored dry for 1, 2, and 3 months. df, degree of freedom; MS, mean square; SS, sum of squares.

Source	df	SS	MS	*F*-Value	*P*-Value
Storage time	3	50320.188	16773.396	509.894	<0.001
Temperature	3	30855.687	10285.229	312.661	<0.001
GA_3_	3	84836.188	28278.729	859.645	<0.001
Storage time × Temperature	9	8252.563	916.951	27.874	<0.001
Storage time × GA_3_	9	8858.063	984.229	29.920	<0.001
Temperature × GA_3_	9	8566.563	951.840	28.935	<0.001
Storage time × Temperature × GA_3_	27	15440.188	571.859	17.384	<0.001
